# Short-term prognosis, mid- and long-term clinical follow-up of bronchoscopic intervention therapy combined with bronchial artery embolization on massive hemoptysis

**DOI:** 10.3389/fsurg.2025.1642449

**Published:** 2025-08-07

**Authors:** Kunying Li, Taomei Lian, Zhenjing Wang, Zhengxun Zhang, Wenbo Wang, Jiaping Qian

**Affiliations:** ^1^ Department of Endoscopy, Henan Provincial Chest Hospital, Zhengzhou, Henan, China; ^2^ First Department of Tuberculosis, Henan Provincial Chest Hospital, Zhengzhou, Henan, China; ^3^ Department of Endoscopy, Henan Provincial Chest Hospital, Zhengzhou, Henan, China; ^4^ Fifth Department of Cardiology, Henan Provincial Chest Hospital, Zhengzhou, Henan, China; ^5^ Third Department of Thoracic Surgery, Henan Provincial Chest Hospital, Zhengzhou, Henan, China; ^6^ Department of Endoscopy, Henan Provincial Chest Hospital, Zhengzhou, Henan, China

**Keywords:** therapeutic response, massive hemoptysis, postoperative complications, bronchoscopic intervention therapy, bronchial artery embolization

## Abstract

**Background:**

To explore the short-term prognosis, mid- and long-term clinical follow-up of bronchoscopic intervention therapy (BIT) combined with bronchial artery embolization (BAE) in the treatment of massive hemoptysis.

**Methods:**

Patients with massive hemoptysis who received BAE (BAE group) or BAE combined with BIT (BAE & BIT group) based on standard clinical treatment were included retrospectively, during June 2022 and March 2023. The short-term prognosis assessed by the therapeutic response, hemoptysis recurrence rates at 1-month post-surgery, and postoperative complications were compared. Mid- and long-term clinical follow-up data were collected.

**Results:**

Data were collected from 136 patients. There were 68 patients (aged 21–60 years, 31 males) received BAE & BIT, and 68 patients (aged 20–59 years, 36 males) received BAE were matched. After surgery, the immediate response rate and total response rate in BIT & BAE group were significantly higher than those in BAE group, while comparable hemoptysis recurrence rates at 1-month post-surgery. Patients in BIT & BAE group had significantly lower rates of postoperative complications, including obstructive pneumonia lung consolidation, and atelectasis, compared to those in BAEgroup. No death in the BIT & BAE group, while the BAE group experienced 3 intraoperative deaths and 4 cases requiring conversion to surgical intervention. No recurrence was observed during the mid-term and long-term follow-up.

**Conclusion:**

Compared to BAE alone, BIT combined with BAT may offer better short-term prognosis, including higher response rate, lower rates of postoperative complications and mortality, in the management of massive hemoptysis, with a good mid- and long-term therapeutic effects.

## Introduction

Massive hemoptysis is a common respiratory emergency in clinical practice, and if not treated promptly, it can lead to asphyxiation, shock, and even death (1) The mortality rate for massive hemoptysis is reported to be over 50% (2). The etiology of massive hemoptysis is complex, and it can be caused by respiratory and systemic diseases (3). Bronchiectasis, pulmonary tuberculosis, pulmonary aspergillosis, and lung cancer are considered the most common underlying reasons for massive hemoptysis. In recent years, the widespread adoption of respiratory endoscopy techniques has led to a significant increase in the incidence of massive hemoptysis related to bronchoscopic interventions (4).

Bronchial artery embolization (BAE) is currently a widely accepted non-surgical treatment method. In recent years, bronchoscopic intervention therapy (BIT) has been applied effectively in the management of massive hemoptysis. Past studies and clinical practice have shown that BAE is effective in treating massive hemoptysis and holds promise as a treatment option. BAE can rapidly and effectively control bleeding, reducing the risks associated with massive hemoptysis, particularly for patients who are unable to tolerate surgical intervention or have a high surgical risk (5). In some cases, BAE can serve as an alternative to traumatic surgery, avoiding surgical risks and reducing patient suffering (6). This technique is applicable to various diseases causing massive hemoptysis, including pulmonary tumors, tuberculosis, atelectasis, and has expanded its clinical applications (7). However, BAE also has some limitations. Some studies suggest a relatively high recurrence rate after BAE, potentially necessitating multiple treatments to maintain hemostasis (8). Additionally, BAE can lead to complications such as atelectasis and bronchial inflammation, which need to be carefully considered (8).

BIT involves the introduction of specific treatment instruments into the respiratory tract using a bronchoscope to directly address abnormalities within the airways (9). This technique can be employed to manage lesions within the lungs, such as tumors, stenosis, ulcers, and bleeding points (10, 11). The advantages of BIT include its minimally invasive nature, which avoids the trauma associated with traditional surgery, and the ability to perform precise localization and intervention under direct visual guidance (11). Combining BIT with BAE allows for comprehensive treatment of massive hemoptysis caused by different locations and etiologies. For example, if there are intrapulmonary lesions like stenosis or bleeding points, they can be directly addressed using bronchoscopic techniques (3). If the primary cause of massive hemoptysis is bleeding from the bronchial arteries, BAE can be performed to block the bleeding source (6). The strength of this combined treatment strategy lies in its high degree of specificity, allowing for the selection of the most suitable treatment method based on the individual case. Nevertheless, further clinical data is needed to support the prognosis of BIT in combination with BAE in the management of massive hemoptysis. Therefore, this study aims to explore the prognosis of BIT combined with BAE in the treatment of massive hemoptysis.

## Methods

### Study design and patients

This respective study included patients with massive hemoptysiscaused by conditions such as pulmonary tuberculosis and bronchiectasis who treated at Henan Provincial Chest Hospital between June 2022 and March 2023.Inclusion Criteria: (1) Adult patients aged 18–60 years. (2) Inadequate or ineffective response to internal medical conservative treatment, uncontrollable hemoptysis, inadvisable for BAE or BIT & BAE surgery, or refusal of surgical intervention. (3) Patients who have provided informed consent and are willing to undergo interventional treatment, with a follow-up duration of at least six months.Exclusion Criteria: (1) Patients with severe bleeding tendencies or coagulation disorders. (2) Those with severe heart and lung dysfunction, comorbidities, or poor baseline health status, making them intolerant of BAE or bronchoscopic examination. (3) Individuals who have not signed the informed consent for the procedure or cannot cooperate with the interventional treatment. All patients received interventional treatment in addition to their standard clinical care. All patients signed informed consent forms and willingly underwent this treatment method. This study has been approved by the Ethics Committee of Henan Provincial Chest Hospital.

### Typical BAE and BIT & BAE protocol

All cases received continuous cardiac monitoring, oxygen supplementation, and emergency endotracheal intubation to establish an artificial airway for mechanical ventilation. The specific procedure for BAE was as follows: Using a modified Seldinger puncture technique, the femoral artery was percutaneously punctured, and an arterial sheath was left in place. A 5F Cobra catheter was introduced via a guidewire to perform angiography of the affected bronchial artery, assessing the vascular condition and determining whether the bleeding artery shared a common trunk with the spinal artery. Once confirmed as the bleeding artery, a 3F coaxial microcatheter was used for ultra-selective catheterization, embolizing the artery to two or more levels below the bronchial artery bifurcation. The selection of embolization materials, such as gelatin sponge, polyvinyl alcohol particles, coil, etc., was based on the diameter of the supplying vessel and the nature of the lesion, and embolization was continued until the affected vessel was no longer visualized. Subsequent arterial angiography was performed to ensure complete occlusion of the artery before searching for other supplying arteries, such as bronchial artery branches, intercostal arteries, and intrathoracic arteries, gradually embolizing all arteries involved in blood supply, ensuring that no blood vessels supplying the spinal cord were compromised. Combining data from chest x-rays or chest CT scans, after complete embolization of the blood vessels supplying the bleeding site, the catheter was removed, and the puncture site was compressed and bandaged. Post-operatively, the patient remained in a supine position for 24 h.

For BIT combined with BAE treatment, the procedure involved simultaneous bronchoscopic examination during BAE to identify the bleeding site and clear blood from the airway. The procedure details were as follows: The flexible bronchoscope was inserted through the endotracheal tube, and blood was suctioned while advancing. Upon reaching the vicinity of the carina, the bleeding source was observed. Initially, the bronchoscope entered the less blood-filled main bronchus on one side, and blood was suctioned while observing the distal bronchial lumen. Subsequently, the bronchoscope entered the main bronchus on the more blood-filled side, with simultaneous suction and observation. If necessary, a 4°C saline lavage was performed under bronchoscopic guidance. Active bleeding was addressed by temporarily placing a balloon catheter to occlude the affected side's bronchus, ensuring that ventilation on the healthy side remained intact. After the successful implementation of BAE, the balloon catheter was removed, and the airway was rinsed, suctioned, and cleared of blood under bronchoscopic guidance. The effectiveness of the BAE procedure was observed, and in cases of continued bleeding, the bleeding site was identified and treated with repeated BAE. After confirming no further bleeding, both main bronchi were thoroughly examined to ensure airway patency, preventing lung collapse and secondary lung infections. The endotracheal tube was removed when the patient's condition stabilized.

**Figure 1:**
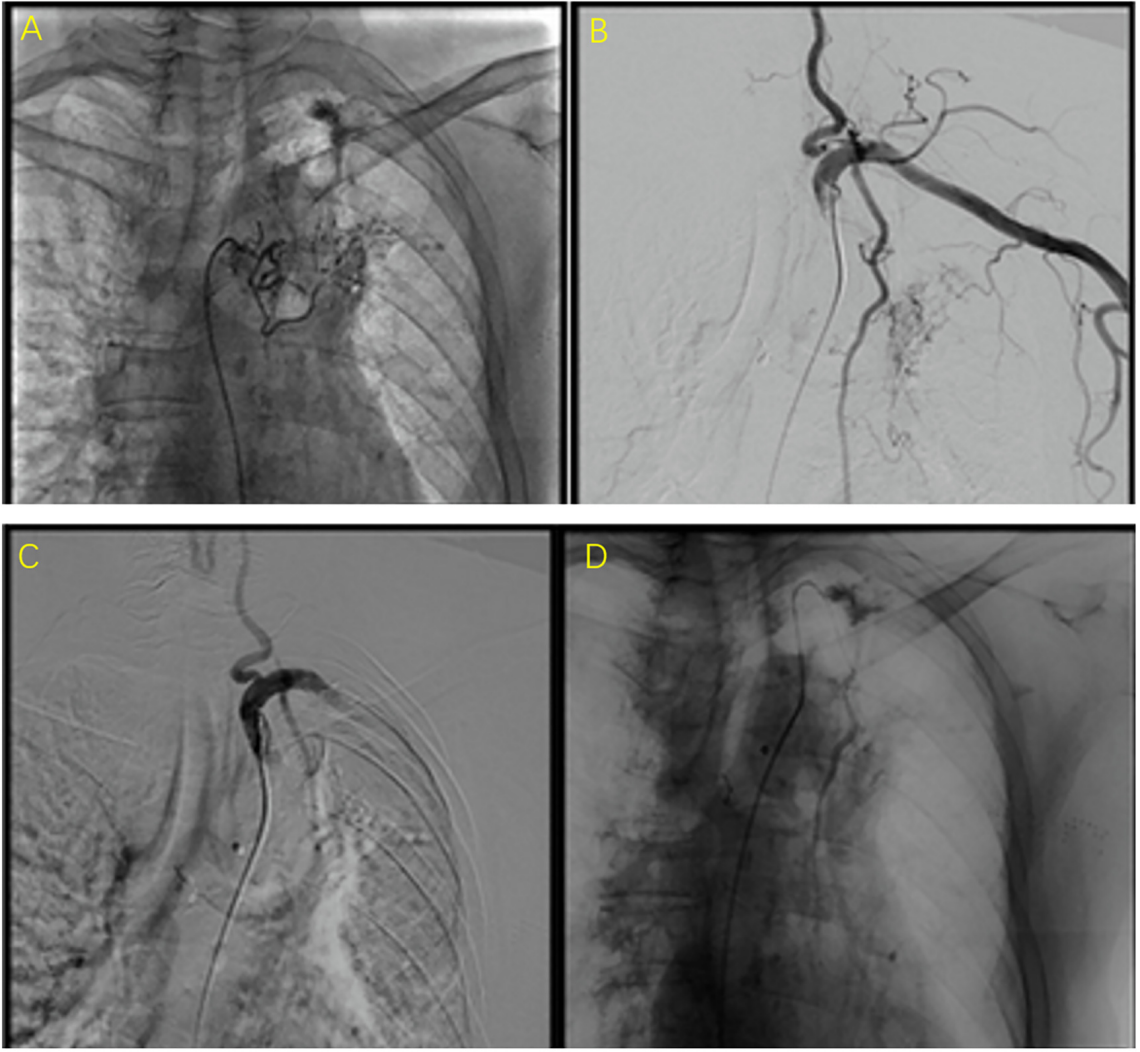
The typical results of the angiography of the responsible vessels before and after embolization of the patients. **(A**,**B)** were images before embolization (i.e., the abnormal conditions observed during the angiography of the responsible vessel). **(C**,**D)** were images after embolization. They present as follows: (1) Direct signs: contrast agent extravasation, pseudoaneurysm; (2) Indirect signs: capillary hyperplasia, abnormal staining of lung tissue, thickening/distortion of the main artery, aneurysm, shunt/drainage between systemic circulation and pulmonary circulation, etc.

**Figure 2:**
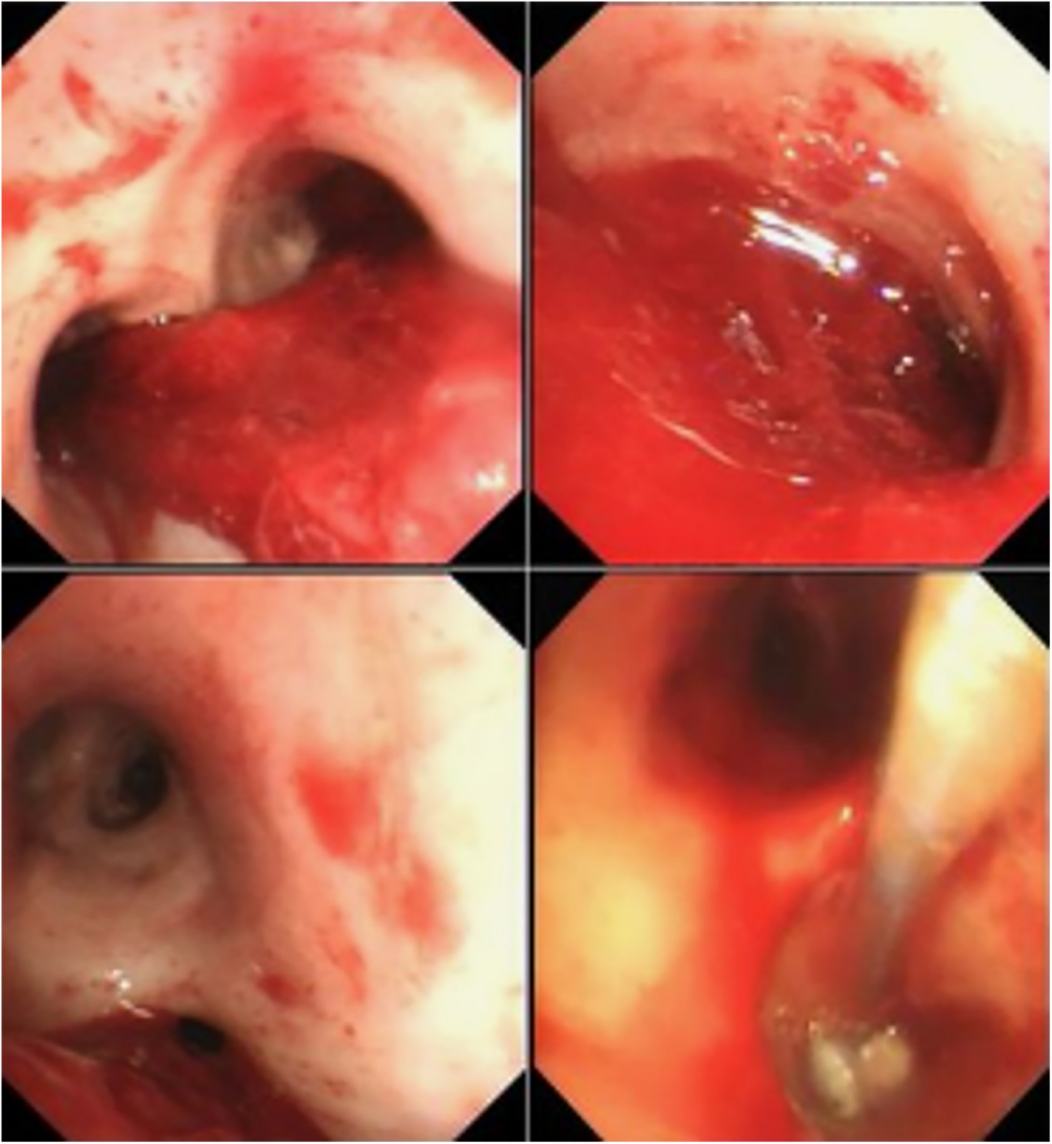
The typical results of bronchoscop. **(A)** A large amount of blood clots could be seen in the lower part of the trachea and the bronchial cavities on both sides, mainly in the left bronchus, and there was also visible bleeding. It was judged that there was bleeding in the left lung. **(B)** A large amount of blood clots was found in the left bronchial cavity, and there is also visible bleeding. **(C)** After removing a considerable amount of blood clots, a large amount of blood clots blocking the bronchial cavity in the lower lobe of the left lung were observed, and there was continuous bleeding. It was judged that there was bleeding in the lower lobe of the left lung. **(D)** The picture of interventional treatment under bronchoscopy, using a balloon to seal the vascular orifice under the bronchoscope to ensure the successful implementation of the BAE procedure.

Basic requirements for BAE intraoperative operations: (1) Standard equipment: A catheterization operating room equipped with digital subtraction angiography (DSA) equipment; (2) Real-time monitoring of blood pressure, heart rate, blood oxygen saturation, and respiratory rate, and the ability to handle asphyxia and shock emergencies; (3) Physician qualifications: According to the “Notice on Clinical Application Management Specifications for 4 Interventional Diagnostic and Therapeutic Techniques Including Cardiovascular Diseases Intervention” issued by the Office of the National Health Commission in Document No. 828 (2019) of the National Health Commission, BAE falls under the category of tertiary surgeries in both comprehensive interventional and cardiovascular disease interventional fields. It should be performed by physicians with the following qualifications: (1) Specialization in medical imaging and radiation therapy or clinical specialties compatible with the implementation of comprehensive interventional diagnostic and therapeutic techniques; (2) Completion of relevant system training for comprehensive interventional diagnostic and therapeutic techniques and passing the assessment; (3) At least 3 years of clinical professional experience in the relevant field and holding a chief physician or above title. The typical results of the angiography of the responsible vessels before and after embolization of the patients were shown in Figures 1A–D. The relevant responsible blood vessels need to be embolized, and the entire embolization process must be carried out under fluoroscopy. The typical results of bronchoscopy are shown in Figures 2A–D.

**Figure 3:**
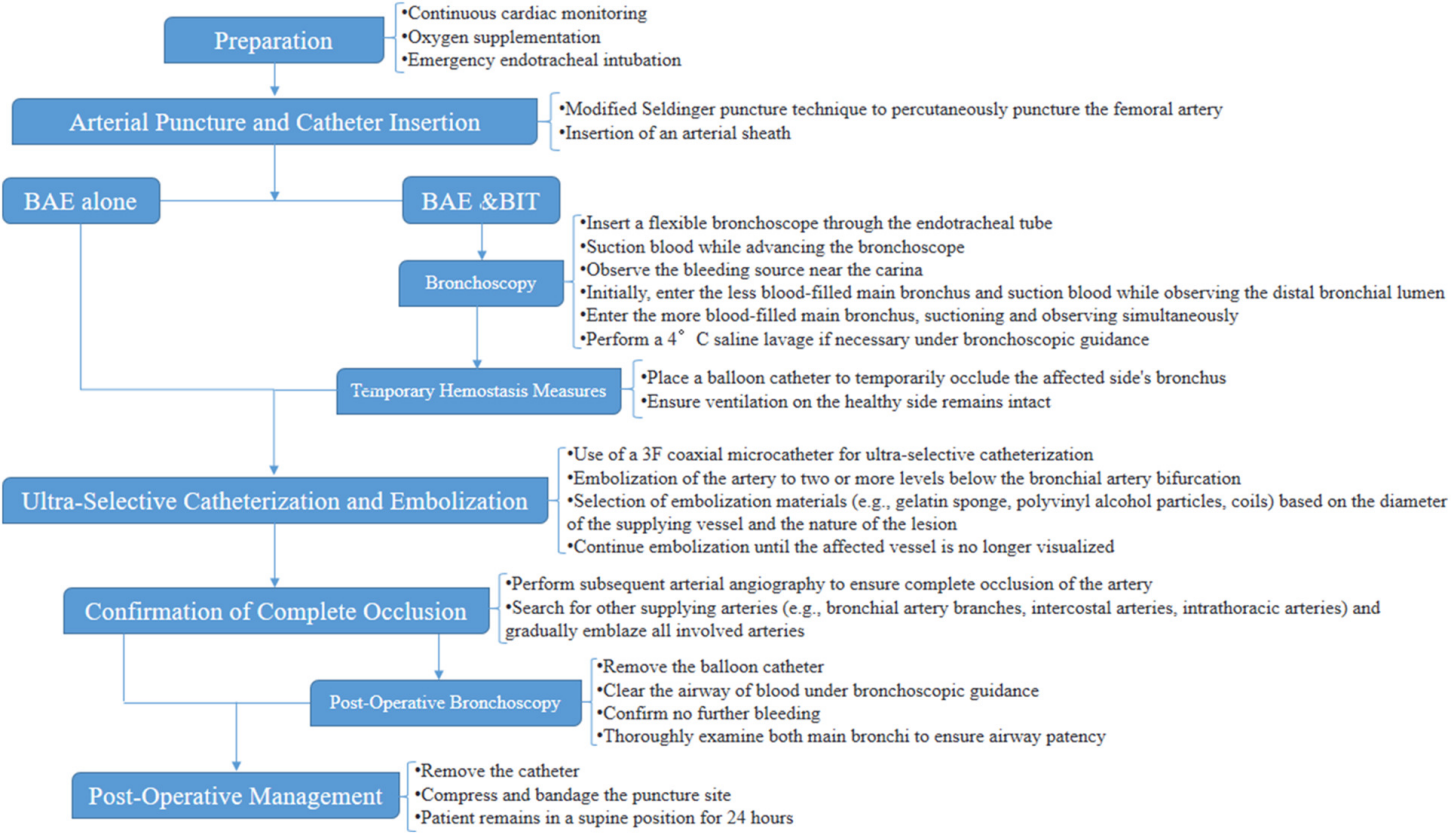
A flowchart for BAE and BIT & BAE protocol.

All patients were given intravenous infusion of pituitary posterior lobe hormone (8–16 u diluted) for 3–5 days after the procedure. Hemoptysis was monitored during the first week post-operation, and follow-up included blood tests, C-reactive protein, procalcitonin (PCT), and high-resolution chest CT to assess the incidence of postoperative complications. A flowchart for BAE and BIT & BAE protocol were showed in Figure 3.

### Data collection and definition

Patients’ clinical data, including gender (male/female), age (years), duration of hemoptysis (days), daily hemoptysis volume (ml), underlying cause of hemoptysis [n (%), pulmonary tuberculosis and hemoptysis or bronchiectasis and hemoptysis], preoperative SaO_2_%, were collected. Moreover, their therapeutic response, the hemoptysis recurrence rates at 1-month post-surgery (BAE and BIT & BAE), andpostoperative complications were recorded. The prognosis was assessed by the therapeutic response, hemoptysis recurrence rates at 1-month post-surgery (BAE and BIT & BAE), and postoperative complications. The therapeutic response was measured by immediately response rate and total response rate. Total response rate was dedined as: (1) Cure: Active hemoptysis stops immediately after embolization or gradually ceases within 3–5 days, and there is no recurrence of hemoptysis. (2) Significant improvement: The frequency of hemoptysis significantly decreases, with only blood-tinged sputum or a reduction of over 90% in the maximum daily hemoptysis compared to before treatment, and it does not affect daily life and work. (3) Effective: The frequency of hemoptysis decreases, and the maximum daily hemoptysis volume is reduced by over 50% compared to before treatment. (4) Ineffective: The above criteria are not met. The postoperative complications including obstructive pneumonia, pulmonary consolidation, and atelectasis. Mid-(3 years) and long-term (5 years) follow-up data were collected.

### Priori power analysis

To address the concern regarding the sample size and its impact on the reliability of our findings, we conducted an *a priori* power analysis to determine the required sample size for detecting a significant difference in the primary outcome measure, which was the immediate response rate. Based on previous studies and clinical experience, we assumed that the immediate response rate would be 95% in the BAE & BIT group and 85% in the BAE group. Using G*Power, we performed a power analysis for two independent proportions with a significance level (α) of 0.05 and a desired power (1-β) of 0.80. The effect size (odds ratio) was calculated based on these assumed response rates. The analysis indicated that a sample size of 60 patients per group would be sufficient to detect the expected difference in the immediate response rate with 80% power.

### Statistical analysis

This study employed SPSS 19.0 (IBM Corp., Armonk, N.Y., USA) statistical software for data analysis. To ensure comparability between the BAE & BIT group and the BAE group, a propensity score matching (PSM) method was employed. The PSM approach was used to balance the distribution of potential confounding variables across the two groups. The following covariates were included in the propensity score model: age, gender, underlying lung disease (e.g., tuberculosis, bronchiectasis), severity of the underlying condition, volume of hemoptysis, and previous treatment history (including anticoagulation therapy and bleeding tendency). The propensity scores were calculated using a logistic regression model with the above-mentioned covariates. A 1:1 nearest-neighbor matching algorithm without replacement and with a caliper width of 0.2 standard deviations of the logit of the propensity score was applied to match patients in the BAE & BIT group with those in the BAE group. After matching, standardized mean differences (SMDs) were calculated for each covariate to assess the balance between the two groups. An SMD < 0.1 was considered indicative of adequate balance. Patients who received BAE & BIT were successfully matched with the same numbers of patients who received BAE alone. The matching process resulted in well-balanced groups with respect to all baseline characteristics, as evidenced by SMDs below 0.1 for all covariates. This rigorous matching procedure ensured that any observed differences in outcomes could be attributed more confidently to the intervention rather than to pre-existing differences between the groups. Continuous data in this study followed a normal distribution and are presented as mean ± standard deviation (mean ± SD). Group-to-group comparisons were conducted using independent sample t-tests. Categorical data were presented as relative numbers (n %), and inter-group comparisons were performed using the chi-squared test (*χ*² test). Two-sided *P*-value less than 0.05 was considered statistically significant.

## Results

Clinical data were collected from 136 patients. There were 68 patients (aged 21–60 years, 31 males) received BAE & BIT, and 68 patients (aged 20–59 years, 36 males) received BAE were matched. The duration of hemoptysis in both groups ranged from 6 days to 1 month, with daily hemoptysis volumes between 100 and 500 ml. Among them, the BAE group had 30 patients with pulmonary tuberculosis and 38 patients with bronchiectasis, while the BAE & BIT group had 32 patients with pulmonary tuberculosis and 36 patients with bronchiectasis. There were no statistically significant differences in gender, age, underlying causes of hemoptysis, or hemoptysis characteristics between the two groups (*P* > 0.05) (Table 1).

**Table 1:** Baseline characteristics.

Characteristics	BAE group (*n* = 68)	BIT & BAE group (*n* = 68)	*P* value
Gender, *n* (%)			0.391
Male	36 (52.9)	31 (45.6)	
Female	32 (47.1)	37 (54.4)	
Age (years), (mean ± SD)	37.4 ± 11.2	41.2 ± 9.6	0.148
Duration of hemoptysis (days), (mean ± SD)	14.7 ± 8.2	16.1 ± 7.7	0.372
Daily hemoptysis volume (ml), (mean ± SD)	178.6 ± 22.3	184.4 ± 27.9	0.117
Underlying cause of hemoptysis, *n* (%)			0.731
Pulmonary tuberculosis	30 (44.1)	32 (47.1)	
Bronchiectasis	38 (55.9)	36 (52.9)	
Preoperative SaO_2_%, (mean ± SD)	86.6 ± 1.2	84.7 ± 1.9	0.213

Patients in BAE & BIT group demonstrated a significantly higher immediate response rate [68 (100%) vs. 61 (89.7%), *P* = 0.007] and overall response rate [68 (100%) vs. 61 (89.7%), *P* = 0.007] compared to those in BAE group, also, with a higher percentage of cures [55 (80.55%) vs. 47 (69.12)]. Additionally, the hemoptysis recurrence rates at 1 month after surgery were comparable [14 (20.6%) vs. 13 (19.1), *P* = 0.830] between the two groups. In addition,the occurrence of postoperative complications in patients receiving the BIT & BAE, including obstructive pneumonia [4 (0.6%) vs. 16 (23.5%), *P* = 0.004], pulmonary consolidation [2 (3%) vs. 13 (19.1%), *P* = 0.003], and atelectasis [0 (0) vs. 11 (16.2%), *P* = 0.001], was significantly lower than in patients treated with BAE alone (Table 2). Furthermore, there were no cases of mortality in the BIT & BAE group, while the BAE group experienced 3 intraoperative deaths and 4 cases requiring conversion to surgical intervention. No recurrence was observed during the mid-term and long-term follow-up.

**Table 2:** Comparison of immediately response rate, total response rate, hemoptysis recurrence rates at 1-month post-surgery, and postoperative complications.

Outcomes	BAE group (*n* = 68)	BIT & BAE group (*n* = 68)	*P* value
Immediately response rate, *n* (%)	61 (89.7)	68 (100)	0.007
Total response rate, *n* (%)	61 (89.7)	68 (100)	0.007
Cure	47 (69.1)	55 (80.6)	
Significant improvement	9 (13.2)	7 (10.3)	
Effective	5 (7.2)	6 (8.8)	
Ineffective	7 (10.3)	0 (0.0)	
Hemoptysis recurrence rates at 1-month post-surgery	14 (20.6)	13 (19.1)	0.830
Postoperative complications			
Obstructive pneumonia	16 (23.5)	4 (6)	0.004
Pulmonary consolidation	13 (19.1)	2 (3)	0.003
Atelectasis	11 (16.2)	0 (0)	0.001

## Discussion

This study demonstrates that BIT combined with BAE may have better short-term prognosis compared to BAE alone, including higher response rate, lower rates of postoperative complications and mortality, in the management of massive hemoptysis. These findings provide support for the clinical application of BIT combined with BAE for the management of massive hemoptysis.

Massive hemoptysis was traditionally considered a contraindication for bronchoscopy (12). However, with the widespread use of bronchoscopy, accumulating clinical experience has led to a narrowing of the relative contraindications (13). In the 2019 Guidelines for Flexible Bronchoscopy in Adults (12), it was suggested that bronchoscopy during active massive hemoptysis was associated with higher risks, and the benefits should be carefully weighed. If bronchoscopy during active hemoptysis is deemed necessary, preparations should be made for establishing an artificial airway and providing emergency care to address the increased risk of suffocation due to worsened bleeding. In recent years, BIT has been applied in the management of massive hemoptysis. This involves procedures such as injecting vasoconstrictors, coagulants, and using balloon tamponade to achieve successful outcomes. A study by Zhang Shengbin et al. (14) retrospectively analyzed 15 cases of successful treatment of massive hemoptysis and acute respiratory failure, emphasizing the importance of maintaining airway patency and oxygenation. In cases of massive hemoptysis, the primary goal is to ensure airway patency, maintain oxygenation, and establish an artificial airway as needed. BIT for removing intrabronchial blood or clots can help prevent suffocation and are life-saving. If conventional pharmacological hemostasis is ineffective or when there is initial hemostasis and endoscopic treatment proves unsuccessful or fails, BAE should be considered as the first-line option (13, 15), given its advantages of minimal invasiveness and good prognosis. In line with the current study, immediate clinical success rates of BAE have been reported to range from 70% to 99% (15). However, surprisingly, the current study reveals that the BIT & BAE group, utilizing BIT, achieved 100% immediate hemostasis and overall effectiveness, which is a significantly higher rate than that seen with BAE alone. This can be attributed to the fact that in the BIT & BAE group, BIT is performed to identify the bleeding site, promptly clear intrabronchial blood, and create a clear airway, ultimately facilitating the success of BAE (16). Specifically, BIT has the following advantages: (1) it allows for rapid and precise operations; (2) during the guided intubation process, blood accumulation in the throat can be promptly cleared, ensuring a clear view of the glottis and increasing the success rate of one-time intubation; (3) a fiberoptic bronchoscope can provide local surface anesthesia, minimizing the direct stimulation caused by the tracheal catheter, making it suitable for critically ill awake patients; (4) the bronchoscope can help clear blood accumulation or clots from deeper parts of the airway during intubation, smoothing the passage; and (5) bronchoscope-guided intubation enables direct observation of the catheter's position, preventing it from straying into one side of the main bronchus or the esophagus. This combination results in better safety and prognosis.

In assessing recurrence, the recurrence rates after BAE have shown significant variation, ranging from 9.8% to 57.5% (15), which is consistent with the 20.6% hemoptysis recurrence rate at one month reported in this study. Importantly, the study shows that the BIT & BAE group also achieved a one-month hemoptysis recurrence rate of 19.1%. This suggests that the choice between the two treatment strategies, BIT and BAE, may not significantly affect the hemoptysis recurrence rate. Several factors may contribute to this observation. (1) Patients with massive hemoptysis might have underlying chronic conditions, such as bronchiectasis, tuberculosis, or lung cancer, which can persist and lead to recurrent bleeding. The addition of BIT, while effective in the short term, may not completely eliminate the underlying pathology that continues to cause bleeding over time. (2) BAE can effectively occlude the target arteries, but the presence of collateral circulation can allow blood flow to resume, leading to recurrent bleeding. BIT, while improving immediate hemostasis, may not fully address the development of new collateral vessels. (3) BIT interventions, such as endobronchial tamponade and clot removal, provide immediate relief but may have a temporary effect. The underlying bleeding sources can re-open or new sources can develop, leading to recurrence. (4) The effectiveness of BIT can be limited by technical challenges, such as difficulty in accessing certain areas of the airways or the presence of extensive bleeding that makes visualization and intervention challenging. (5) The postoperative care and follow-up protocols may differ between patients, affecting the recurrence. Consistent and rigorous follow-up, including regular monitoring and timely intervention, is crucial for preventing recurrence. (6) Patient compliance with postoperative care instructions, such as medication adherence and lifestyle modifications, can vary and impact the recurrence rates. (7) The relatively small sample size of our study may have limited the statistical power to detect a significant difference in recurrence rates. Larger studies with longer follow-up periods may be needed to more accurately assess the long-term benefits of the combined therapy. In addition, a systematic review has reported that hemoptysis recurrence after BAE may be due to incomplete embolization, recanalization of previously embolized arteries, or new collateral artery recruitment caused by the progression of underlying diseases (15). Li H et al. (17) found that treatment of underlying pulmonary diseases and comorbidities is crucial for improving BAE success rates and reducing the risk of hemoptysis recurrence after BAE.

In situations where the etiology of massive hemoptysis is unclear, or when conservative medical treatment for hemostasis is ineffective, an early bronchoscopic examination has been advocated (9). The primary goal in managing massive hemoptysis is to ensure airway patency and isolate the bleeding source (18). During active bleeding, the most effective way to clear the airway is through the patient's own cough reflex. If patients cannot expel blood or develop progressive respiratory distress or hypoxemia, emergency endotracheal intubation to establish an artificial airway is crucial. In such cases, BIT, involving the removal of intrabronchial blood or clots, is used to maintain a patent airway, preventing suffocation. The present study utilized a combined approach with BIT and BAE, which resulted in no cases of mortality and significantly reduced the incidence of postoperative complications, including obstructive pneumonia, pulmonary consolidation, and atelectasis. The reasons could be BIT enables the removal of blood clots and the application of hemostatic agents, reducing the risk of mucus plugging and subsequent infections. The combination of BAE and BIT provides a dual mechanism of hemostasis, ensuring more complete and sustained control of bleeding and minimizing the duration of active bleeding. Moreover, BIT allows for targeted management of airway lesions, preventing further complications. This underscores the importance of bronchoscopy as a valuable tool in the diagnosis and management of massive hemoptysis. BIT helps to identify the site of bleeding, administer localized hemostatic agents, use balloon tamponade for hemostasis, and isolate the source of bleeding, all of which buy time for the successful implementation of BAE (16) After BAE, bronchoscopic examination allows for direct observation of the therapeutic effect and removal of residual intrabronchial blood, ensuring the patency of bronchial lumina and preventing postoperative complications.

This study has limitations. Firstly, it is a single-center retrospective study, which may introduce selection bias and may not represent the characteristics and situations of a broader population. Secondly, the sample size is small, and no multi-factor correction was performed. As a result, the statistical analysis may lack power and reliability. Thirdly, due to the retrospective nature of the study, the researchers could not control the manner and timing of data collection. This could lead to incomplete or erroneous data, and it may not capture long-term follow-up data for patients. Nonetheless, this study provides further analysis and support for the clinical application of BIT combined with BAE in the treatment of massive hemoptysis.

## Conclusion

In conclusion, compared to BAE alone, BIT combined with BAT may offer better short-term prognosis, including higher response rate, lower rates of postoperative complications and mortality, in the management of massive hemoptysis, with a good mid- and long-term therapeutic effects. However, further prospective, large-scale, multi-center clinical studies are necessary to confirm these findings. Additional recommendations for future studies included: (1) Evaluate the efficacy in patients with varying underlying conditions and severities of hemoptysis. (2) Assess long-term recurrence rates, quality of life, and overall survival. (3) Refine procedural techniques and standardize treatment protocols. (4) Investigate the combination of BAE, BIT, and other therapies.

## Data Availability

The original contributions presented in the study are included in the article/Supplementary Material, further inquiries can be directed to the corresponding author.
